# Two-Dimensional Damage Localization Using a Piezoelectric Smart Aggregate Approach—Implementation on Arbitrary Shaped Concrete Plates

**DOI:** 10.3390/ma17010218

**Published:** 2023-12-30

**Authors:** Nemanja Marković, Dušan Grdić, Nenad Stojković, Gordana Topličić-Ćurčić, Darko Živković

**Affiliations:** 1 Department for Materials and Structures, Faculty of Civil Engineering and Architecture, University of Niš, 18000 Niš, Serbia; dusan.grdic@gaf.ni.ac.rs (D.G.); gordana.toplicic.curcic@gaf.ni.ac.rs (G.T.-Ć.); darko.zivkovic@gaf.ni.ac.rs (D.Ž.); 2 The Academy of Applied Technical and Educational Studies, University of Niš, 18000 Niš, Serbia; svnenad@yahoo.com

**Keywords:** structural health monitoring, damage detection, piezoelectric smart aggregates, concrete, wave propagation, damage localization, finite element method, reinforced concrete structures

## Abstract

This paper presents the application of a hybrid approach for damage localization in concrete plates of arbitrary geometric shapes and a constant thickness. The hybrid algorithm utilizes fast discrete wavelet transformation, energy approach and time of flight criteria for the purpose of the localization of single- and multi-damage problems inside or on the periphery of concrete plates. A brief theoretical background of the hybrid method as well as numerical procedures for modeling the piezoelectric smart aggregate and ultrasonic wave propagation are presented. Experimental and numerical verification of the damage localization were performed on square samples/models with one or two damages and with 16 positions of piezoelectric smart actuator/sensor aggregates. After the verification of the hybrid method, a numerical simulation was performed on models with one or two damages for plates of arbitrary geometric shapes. Based on the obtained results, it was concluded that the proposed method can be applied to damage localization in concrete plates of arbitrary geometric shapes. The presented method and numerical procedure can be further used in research through varying the geometry, number and position of damages as well as the number and position of piezoelectric smart aggregates.

## 1. Introduction

Structural health monitoring (SHM) represents the integration of sensors and, if necessary, actuators in order to detect, analyze, localize and predict various impacts and damages to the structure in such a way that non-destructive testing and evaluation (NDT/E) become an integral part of the structure and materials [1]. Moreover, SHM implements sensors and actuators, smart materials, data transmission and computer-software potentials alongside the structure in order to detect, localize, quantify and assess the damage that can affect the collapse of the structure at a given moment or over time [2,3]. With applications in civil engineering [4,5,6,7], the aerospace industry [8,9,10,11,12], mechanical engineering [13,14] and the naval industry [15,16,17], SHM represents an interdisciplinary scientific field.

Civil engineering structures are designed to meet the criteria of safety, reliability, durability, serviceability and sustainability throughout the exploitation life of the structure. In order to satisfy all these criteria, it is necessary to have reliable data on the basis of which it is possible to define the characteristics of the materials, the real intensities of the actions, the state of the structure and other influences during the exploitation of the structure as accurately as possible. Monitoring the state of old reinforced concrete (RC) buildings, especially the ones subject to earthquakes, is a huge challenge for researchers [18,19,20].

In the field of SHM, damage is a break in the material continuum that can appear as an imperfection, defect or crack that leads to yielding (failure), i.e., to a change in the functioning and performance characteristics of the structure. In the most general sense, damage can be understood as a change in the structure that affects the current or future functionality of the object or individual element of the structure. In this sense, damage separates two states of the structure presented as follows: (1) the initial state, which is often considered undamaged (in a general sense, it is not always the case) and (2) the damaged state of the structure [21]. The detection, localization and quantification of initial damage, especially of cracks which are the most common type of damage in RC structures, represent a special challenge. A crack can be seen as a resulting material discontinuity of the structure and is most often the result of a local material fracture.

The following SHM methods are used for the detection of damage in structures: (1) a vibration-based method [22,23]; (2) a method based on the application of ultrasonic waves [24,25,26,27]; (3) a method based on electrical impedance [28,29,30]; (4) eddy current—EC [31]; (5) impulse-echo [32,33,34]; (6) acoustic emissions [35,36]; (7) a method based on thermal images [37]; and (8) a method based on the analysis of digital photographs [38,39]. In this research, a method based on ultrasonic wave propagation was applied. Damage detection determines the existence of damage in the structure without detailed information about its location. The next step in SHM is damage localization and quantification. Different tools and methods were used to localize damage in plate structures (thin aluminum, metal or composite plates in the airplane industry) in order to develop SHM methods that can localize damage in practical conditions. However, many of these methods are not applicable to damage localization in concrete plates (which do not have negligible thickness), where currently there is not a large amount of research. The first methods used the reconstruction of signals obtained based on the reception of an incoming ultrasonic wave with PZT sensors. When analyzing signals, time of flight—ToF and delay and sum—DAS are very often used as criteria for developing algorithms based on which the position of the damage is obtained. One example of such research is the use of the difference in time of propagation of waves (TDOA) and DAS in combination for damage localization in concrete structures [40]. Moreover, the technique for forming damage images based on the local phase differences of wave propagation speeds has been successfully utilized using transverse waves to localize material imperfections [41]. The accuracy of methods in damage localization and quantification varies from highly accurate to those that can only determine the zones where the damage is located. Certain methods are limited to the detection of only one damage, others have certain “blind zones”, while others “suffer” from the poor precision and quality of the obtained damage images. This paper presents an original hybrid method for damage localization that can be applied to concrete plates of arbitrary shapes.

The development of SHM methods can be verified experimentally and/or numerically. Experimental research requires significant financial resources, a large amount of time for the preparation and implementation of the experiment and is limited to a relatively small number of samples. Numerical simulations can significantly speed up the development of methods because different model geometries, positions and numbers of PZT sensors/actuators and other parameters can be varied relatively quickly. Several different methods were used to model wave propagation including (1) a boundary element method [42]; (2) a finite difference method [43]; (3) a global matrix method [44]; (4) a mass-spring lattice method—MSLM [45]; (5) a mesh-less method—MLM [46]; (6) a local interaction simulation approach—LISA [47]; (7) and a mesoscale finite element method [48] in order to find the most efficient way to model wave propagation. Furthermore, the application of the FEM in combination with experimental modal analysis (EMA) can be effective in vibration monitoring and control as well as in the dynamic analysis of structures [49,50]. The most common numerical approach for modeling wave propagation is certainly the finite element method (FEM). In a general sense, there are two approaches for the direct time integration of FEM solutions for dynamic problems which are as follows: the implicit FEM (IFEM) [51,52] and the explicit FEM (EFEM) [53,54]. Both integration methods have their advantages and disadvantages. For example, the implicit method requires more time to calculate each individual time step compared with the explicit method. On the other hand, the IFEM for linear problems can have unconditional solution stability so the time increment can be chosen based on the problem being solved, which is not the case with the EFEM. However, the EFEM should be preferred for modeling ultrasonic wave propagation. In this paper, the IFEM was used for modeling PZT SAs, while the EFEM was applied to modeling ultrasonic wave propagation.

Piezoelectric smart aggregates (PZT SAs) are multifunctional devices that, among other things, are used for the damage detection and localization in reinforced concrete structures. A lead zirconate titanate (PZT) patch, protected using a watertight layer and embedded in a very small concrete block, represents the PZT SA. The fabricating process of PZT SAs mainly consists of the following steps: 1. the selection of the size and shape of a PZT patch; 2. the soldering of an electric cable to the PZT patch; 3. the connecting of the electric cable to the BNC connector; 4. the waterproofing of the PZT patch; 5. the making of the formwork with an adequate size and shape and the fixing of the PZT patch in the formwork; 6. the pouring of fresh cement paste. PZT patches are characterized by high brittleness and a lack of resistance to moisture and because of this they were unsuitable for usage in reinforced concrete structures. PZT SA protects the PZT patch from moisture and mechanical shocks during the pouring of fresh concrete and during the building service. A PZT SA is installed into a real structure by fixing it to the reinforcement bars or formwork at a predefined point. Piezoelectric smart aggregates proved to be multifunctional components which can be applied to different purposes in civil engineering [55] such as the monitoring of impact forces of the vehicle on the bridge [56], the monitoring of the connection failure between the concrete and rebars [57], the detection of reinforcement damage inside the reinforced concrete element [58], the monitoring of humidity change in the concrete [59], the vibration control in civil engineering structures [60], the derivation of early concrete strength in city [61] and the derivation of the compression stress during seismic action [62]. The rationality of the application of PZT SAs is reflected in the following characteristics of low cost, active sensing, quick response, availability in different shapes and simplicity of implementation [63]. Extensive experimental research has been carried out on the application of PZT SAs for the detection of damage in beam RC elements, columns and walls under static, quasi-static or dynamic loads [64,65]. The results of this research suggest that PZT SAs can be widely used in the monitoring of construction facilities.

The motivation for conducting this study comes from the three points as follows: 1. based on the review of the available relevant literature, there is a very small amount of research that deals with methods for the detection and localization of damage in concrete slabs [66,67,68] and most of the research has been focused on thin aluminum or steel plates; 2. the author’s previous research, which was based on square-shaped slabs, was extended to the general shape of concrete slabs in order to obtain results that can be used for different situations of combining the following parameters of the shape and dimensions of the concrete slabs, position, number and form of damage as well as the number and location of PZT SAs; 3. finally, the ultimate goal of the work is the use of the hybrid method in the detection and localization of damage in real RC constructions.

The paper is organized into six sections. Section 1 describes the current state of research, various methods for the detection, localization and characterization of the damage. The damage localization hybrid procedure using fast discrete wavelet transformation (FDWT), energy approach (EA) and time of flight (ToF) criteria is presented in Section 2. Numerical modeling of piezoelectric smart aggregates (PZT SAs) using the IFEM and modeling of wave propagation using the EFEM is presented in Section 3. Section 4 of the paper shows the experimental and numerical verification of hybrid approach in cases of square concrete samples/models with one or two damages. Furthermore, the application of the hybrid algorithm for damage localization in irregularly shaped concrete plates with a constant thickness is presented in Section 5. Finally, the conclusion with the analysis of the obtained results and a proposal for future research are given in Section 6.

## 2. Hybrid Approach for Two-Dimensional Damage Detection and Localization

In this section, a hybrid algorithm for damage detection and localization in concrete plates, developed by the author of this paper, will be presented. The algorithm was developed for the general shape of the concrete plate. The approach is referred to as hybrid because it implements three criteria (energy approach, time of flight and discrete wavelet transform of the signal) in the decision-making process of selecting relevant actuator/sensor directions for damage localization. The time of flight (ToF) criterion is based on the fact that the incoming wave from the actuator (A) to the sensor (S) will not be the same for an undamaged structure compared with a damaged concrete element along the direction of the PZT A/S. The energy criterion is defined in Equation (1) and is calculated for all the directions that previously met the ToF criterion. A more detailed procedure for obtaining all the parameters for the application of Equation (1) can be found in [69]. The third last criterion, discrete wavelet signal transformation, is characterized by the fact that the output signal of the sensor can be transformed into the approximation and detail.

(1) $$DI=\sqrt{\frac{\sum_{k=1}^{q}{\left(E_{i,k}-E_{h,k}\right)}^{2}}{\sum_{k=1}^{q}{\left(E_{h,k}\right)}^{2}}}$$

where DI—is the one-dimensional damage index based on the root-mean-square deviation, $E_{i,k}$—is the energy vector for damaged concrete structures, $E_{i,k}$—is the energy vector for undamaged concrete structures. The value of the DI = 0 means that the structure is undamaged and the DI > 0 means that structure has damage inside. A higher DI indicates greater damage.

**Figure 1:**
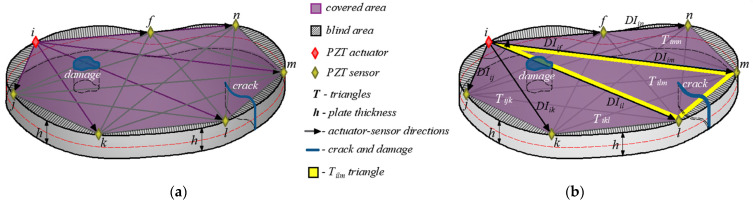
(**a**) PZT SA A/S positions and directions in a damaged concrete plate of arbitrary shape; (**b**) division of concrete plate into triangles in a counter-clockwise direction.

The hybrid algorithm was developed for the general shape of the concrete plates, the arbitrary position and the number of the PZT A/S (with certain limitations) and the arbitrary shape, position and size of damage. A part of the hybrid algorithm concept is shown Figure 1. Damage localization is possible within the area of the PZT A/S bounded by marked nodes (i, j, k, l, m, n and f). Above this surface is the so-called “blind zone”, i.e., a part of the surface concrete plate where there is no information about the damage. The procedure requires a minimum of three PZTs A/S. However, in reality, approximately 8–16 PZTs A/S are required for obtaining a better damage picture.

The damage localization procedure involves the activation of one PZT smart aggregate as an actuator (red rhombus in Figure 1b) which causes the propagation of mechanical waves, while all other PZT SAs are used as sensors (yellow rhombus in Figure 1b). After that, the next one in the counter-clockwise direction is used as an actuator and the others as sensors. The procedure is repeated until the last free PZT SA is used. Consequently, a network of all the directions of the PZT A/S is formed, shown in Figure 1a. For a larger number of A/S, a large number of possible mutual directions are obtained, which can greatly burden the damage localization procedure. Therefore, only the directions that meet all three criteria are followed until the end, while all the other directions are ignored.

The area covered (purple area in Figure 1) by damage localization is further divided into a final number of triangles obtained through connecting the actuator with the surrounding sensors. Figure 1b shows the yellow triangle obtained from actuator (*i*) and sensors (*l*) and (*m*). The division into triangles was made due to the numerical interpolation of damage index values. For PZT sensors that lie in the same direction, only the furthest sensor is taken into account (nodes *i*, *f* and *n* in Figure 1b).

For all the considered A/S pairs, the sensor output signals for the 2D intact plate are calculated (or measured in the case of experimental research) and the results represent the initial reading. After that, the procedure of reading output signals is carried out for damaged models. The first evaluated criterion that is applied on the output signals is ToF, i.e., whether the longitudinal waves travel time difference (Δt) between the actuator and the sensor for the undamaged ($t_{int}$) and damaged ($t_{dem}$) construction exists. If this condition is met, then the signal is further decomposed using discrete wavelet signal transform. In order to practically apply the ToF criterion, the following conditions have to be met whereby (1) the measurements have to be made on the same concrete structure; (2) the external weather conditions have to be relatively equal (temperature, air humidity); (3) the internal humidity of the concrete structure has to be the same as in the initial measurement; (4) the external vibrations should not have a major impact on the output signal (the noise is negligible in relation to the signal energy). After that, for all the A/S pairs that satisfy the ToF criterion, the energy criterion and the discrete wavelet decomposition (DWT) are applied and the DI is calculated. Only those directions that have different energy measurements for the undamaged and damaged states satisfy both criteria. In this part of the method, the assumption is made that the change in the energy of wave propagation occurred due to the occurrence of damage inside the concrete structure without the influence of external factors. The obtained DI for A/S pairs are entered into the matrix $M_{DI}$ given in Equation (2). The value of the matrix elements of the diagonal Matrix elements (*i* = *j*) is equal to 0, while other values are being calculated.

(2) $$M_{DI}=\left[\begin{array}{cccccc} DI_{11} & DI_{12} & \cdots & DI_{1j} & \cdots & DI_{1n}\\ DI_{21} & DI_{22} & \cdots & DI_{2j} & \cdots & DI_{2n}\\ \vdots & \vdots & \ddots & \vdots & \cdots & \vdots \\ DI_{i1} & DI_{i2} & \cdots & DI_{ij} & \cdots & DI_{in}\\ \vdots & \vdots & \cdots & \vdots & \ddots & \vdots \\ DI_{n1} & DI_{n2} & \cdots & DI_{nj} & \cdots & DI_{nn} \end{array}\right]$$

**Figure 2:**
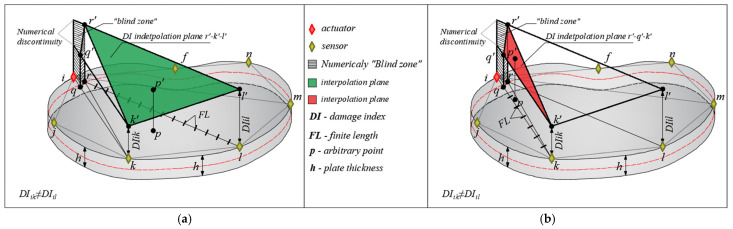
Parts of the linear interpolation plane as follows: (**a**) plane *r’*-*k’*-*l’* and (**b**) plane *r’*-*q’*-*k’*.

**Figure 3:**
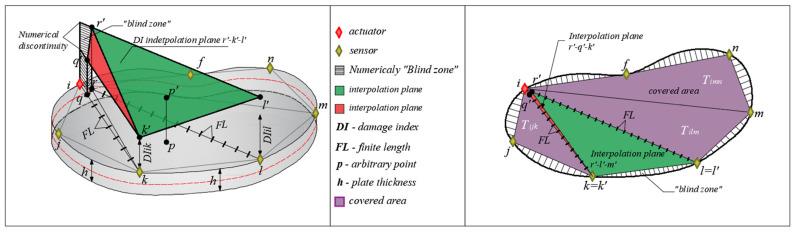
Linear interpolation of the DI over the quadrilateral q-r-k-l.

Then, a linear interpolation of the DI is carried out within the covered surface of the concrete element. The procedure is presented at the level of one triangle. Figure 2 and Figure 3 show the DI interpolation procedure at the level of the triangle $T_{ikl}$. The assumption that the DI is constant along the direction A/S was adopted. Then, the A/S directions were divided into a certain number of final lengths (FL), see Figure 2a. Since the values of the DI are constant along the entire A/S direction and that, in general, they have different values for different directions, the discontinuity of the damage index value in Figure 2 occurs in the actuator node. In order to overcome this problem, new control points are introduced into the calculation of *q* (along the direction *i*–*k*) and *r* (along the direction *i*–*l*) that correspond to the distance FL from node *i*. Furthermore, interpolation of the DI is undertaken on the body *q*-*r*-*k*-*l* instead of the triangle *i*-*k*-*l*. The numerical blind zone *i*-*q*-*r* can be practically ignored due to the great proximity of the actuator. In the case of damage in that zone, there would certainly be a real difficulty in the functioning of the applied actuator.

The damage index values for directions *i*-*k* and *i*-*l* were applied perpendicularly to the plane of the concrete plate in the generated discrete points; new points *q’*, *k’*, *r’* and *l’*, shown in Figure 2, were formed. These four points create two interpolation planes of the damage index. The plane formed by the points *r’*-*k’*-*l’* (green surface in Figure 2a) and the plane *q’*-*k’-r’* (red surface in Figure 2b). These two planes are connected along the r’-k’ line and interpolate the values of the damage index on this part of the plate. It is now possible to determine the damage index for an arbitrarily defined point *p* within the observed part of the concrete surface. Finally, after calculating the DI for all A/S directions and their combinations, at the discrete point *p* the value of the damage index is expressed by the following equation:

(3) $$DI_{p}=\frac{\sum_{z=1}^{s}DI_{z}}{s}$$

where $\sum_{s=1}^{s}{DI}_{s}$ is the sum of the damage index for the selected point for all A/S pairs, *s*—number of summation combinations.

For the hybrid algorithm of damage detection and localization, original MATLAB R2019a codes were created that apply the described procedure. The first MATLAB code performs the discrete wavelet decomposition of the sensor signal and the energy criterion calculation (DI calculation). The second one is made to apply linear interpolation of the DI for all A/S pairs and to create a damage image.

## 3. Numerical Modeling of PZT SA Actuator and Wave Propagation

In this section of the paper, numerical modeling of a PZT SA actuator using the implicit FEM and modeling of ultrasonic wave propagation using the explicit FEM will be presented.

### 3.1. Numerical Modeling of PZT SAs

**Figure 4:**
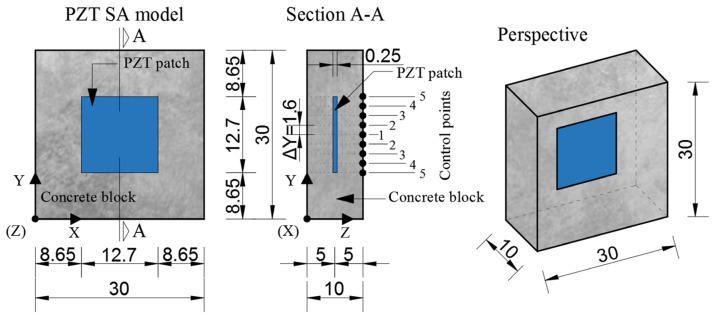
Geometric characteristics of a PZT SA model (dimensions are in mm).

Modeling of piezoelectric smart aggregates (PZT SAs) was performed in the ABAQUS/STANDARD 6.11 software package. Numerical simulation of PZT SAs was performed on a three-dimensional model using the standard finite element method and quasi-static analysis. The smart aggregate model consists of two parts. 1. A piezoelectric patch with dimensions 12.7 × 12.7 × 0.25 mm and a small concrete block (30 × 30 × 10 mm), shown in Figure 4.

The FE modeling of piezoelectric patches is based on constitutive equations of the electromechanical behavior of PZT materials (Equations (4) and (5)).

(4) $$\begin{array}{cc} \sigma_{ij}=D_{ijkl}^{E}\varepsilon_{kl}-e_{mij}^{\varphi }E_{m}, & \begin{array}{c} \;q_{i}=e_{ijk}^{\varphi }\varepsilon_{jk}+D_{ij}^{\varphi (\varepsilon )}E_{j} \end{array} \end{array}$$

(5) $$\begin{array}{cc} \varepsilon_{ij}=S_{ijkl}^{E}\sigma_{kl}-d_{mij}^{\varphi }E_{m}, & \begin{array}{c} \;q_{i}=d_{ijk}^{\varphi }\sigma_{jk}+D_{ij}^{\varphi (\sigma )}E_{j} \end{array} \end{array}$$

with the following notation of $\sigma_{ij},\;\varepsilon_{kl}$—mechanical stress and strain tensor, $q_{i}$—electric “displacement” vector, $D_{ijkl}^{E}$—material elastic matrix defined at zero electrical potential gradient (short circuit condition), $e_{mij}^{\varphi }$—piezoelectric stress coefficient matrix, $d_{mij}^{\varphi }$—piezoelectric strain coefficient matrix, φ—electrical potential, $D_{ij}^{\varphi (\varepsilon )}$—material’s dielectric property, $E_{j}$—electric potential gradient vector.

The PZT patch reacts to the applied electrical voltage (actuator effect) through performing mechanical deformation and vice versa. The electrical voltage is created due to applied mechanical stress (sensor effect). This relationship between electrical voltage and mechanical displacement is a property of piezoelectric materials. In the numerical modeling of PZT SAs, only the first piezoelectric effect—the actuator effect (Equation (5))—was used. Based on Equation (5), it is obvious that the electro-mechanical properties of PZT SAs depend on the dielectric property $D_{ij}^{\varphi (\sigma )}$ and the matrix of piezoelectric coefficients $d_{mij}^{\varphi }$. The PZT patch in this research in the mechanical sense is treated as a linear elastic model. The material model of the piezoelectric patches used in this research is represented by Equations (6)–(8) and Table 1. The orthotropic model of the dielectric matrix $D_{ij}^{\varphi (\varepsilon )}$ was applied. The piezoelectric characteristics of the material were defined through the piezoelectric strain coefficients $d_{ijk}^{\varphi }$. The mechanical characteristics were defined using engineering constants taking into account the transverse isotropy of PZT ferroelectric ceramic materials.

(6) $$\left[\varepsilon \right]=\left[\begin{array}{ccc} D_{11}^{\varphi \left(e\right)} & D_{12}^{\varphi \left(e\right)} & D_{13}^{\varphi \left(e\right)}\\ D_{21}^{\varphi \left(e\right)} & D_{22}^{\varphi \left(e\right)} & D_{23}^{\varphi \left(e\right)}\\ D_{31}^{\varphi \left(e\right)} & D_{32}^{\varphi \left(e\right)} & D_{33}^{\varphi \left(e\right)} \end{array}\right]$$

(7) $$\left[d\right]=\left[\begin{array}{ccc} d_{111}^{\varphi } & d_{122}^{\varphi } & \begin{array}{cccc} d_{133}^{\varphi } & d_{112}^{\varphi } & d_{113}^{\varphi } & d_{123}^{\varphi } \end{array}\\ d_{211}^{\varphi } & d_{222}^{\varphi } & \begin{array}{cccc} d_{233}^{\varphi } & d_{212}^{\varphi } & d_{213}^{\varphi } & d_{223}^{\varphi } \end{array}\\ d_{311}^{\varphi } & d_{322}^{\varphi } & \begin{array}{cccc} d_{333}^{\varphi } & d_{312}^{\varphi } & d_{313}^{\varphi } & d_{323}^{\varphi } \end{array} \end{array}\right]$$

(8) $$\left\{\begin{array}{c} \varepsilon_{11}\\ \varepsilon_{22}\\ \varepsilon_{33}\\ \gamma_{12}\\ \gamma_{13}\\ \gamma_{23} \end{array}\right\}=\left[\begin{array}{cccccc} 1/E_{1} & -\nu_{21}/E_{2} & -\nu_{31}/E_{3} & 0 & 0 & 0\\ -\nu_{12}/E_{1} & 1/E_{2} & -\nu_{32}/E_{3} & 0 & 0 & 0\\ -\nu_{13}/E_{1} & -\nu_{23}/E_{2} & 1/E_{3} & 0 & 0 & 0\\ 0 & 0 & 0 & 1/G_{12} & 0 & 0\\ 0 & 0 & 0 & 0 & 1/G_{13} & 0\\ 0 & 0 & 0 & 0 & 0 & 1/G_{23} \end{array}\right]\left\{\begin{array}{c} \sigma_{11}\\ \sigma_{22}\\ \sigma_{33}\\ \sigma_{12}\\ \sigma_{13}\\ \sigma_{23} \end{array}\right\}$$

A small concrete block is modeled as an isotropic linear elastic material with properties given in Table 1. The contact between the concrete block and the PZT patch is defined through a surface “tie” boundary condition, simulating the full contact of these two elements. The surface of the PZT patch is defined as the “master” surface, while the surface of the concrete block is defined as the “slave” surface. Boundary conditions were defined for the outer edge nodes where the displacement in all three directions was prevented. On one side of the PZT patch an electric voltage equal to zero is fixed, while on the other side a surface electric voltage of 10–100 V with a step of 10 V is applied. The finite element mesh was made using the “sweep” meshing technique with an approximate finite element size of 0.03 mm. A standard linear 3D stress FE C3D8R–8-node linear block with reduced integration and “hourglass” control was used to model the concrete block. For modeling the PZT patch, a standard linear piezoelectric FE C3D8E–8-node linear piezoelectric block was used. The calculation was undertaken with the application of parallel processing.

**Figure 5:**
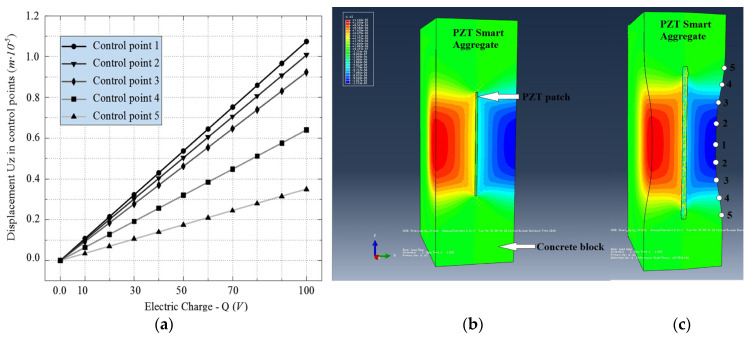
Results of PZT SA numerical model. (**a**) Relation between electric charge *Q(V)* and displacement *U_z_* in control points; (**b**) undeformed PZT SA model; (**c**) deformed PZT SA model from ABAQUS/STANDARD.

**Figure 6:**
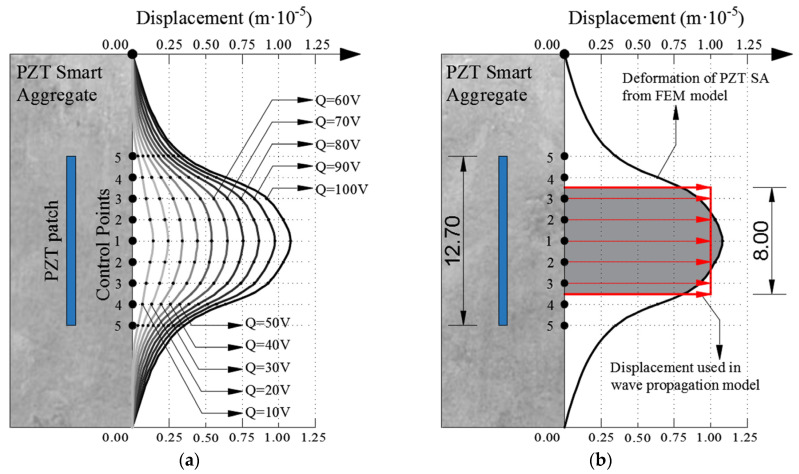
Displacement of PZT SAs. (**a**) Displacement curves for different electric charges; (**b**) approximation form of displacement for modeling wave propagation in ABAQUS/EXPLICIT (lengths in cm).

Figure 5 shows the control points where displacements in the “Z” direction were measured for all the intermediate steps of the applied electric voltage. The results obtained based on the FEM simulation of PZT SAs are shown in Figure 5 and Figure 6. Figure 5a shows the displacements obtained in the control points as a function of the applied electric voltage. One can clearly see the linear dependence between the electric voltage and the resulting displacements. Furthermore, the largest displacement is obtained at control point one which decreases non-linearly towards point five. This can be clearly seen in Figure 5c. Figure 5b,c show the displacements for the undeformed and deformed shape of PZT SAs in order to illustrate the deformation shape of the model.

Figure 6 shows the obtained displacements in the control points based on the FEM model. Figure 6a shows the displacements as a function of electrical voltage where it can be seen that there is a linear dependence between the displacement and electrical voltage. With an increasing electric voltage, the deformation of PZT SAs increases. The deformation of PZT SAs at an electric voltage of 100 V (thicker black line) is shown in Figure 6b. The displacements obtained at this voltage state were used as an input parameter for modeling wave propagation. In order to avoid modeling the boundary conditions of the parabolic displacement (thick black line in Figure 6b), the approximation of the constant intensity marked by the red rectangle in Figure 6b was made. This modeling principle was applied because there is no piezoelectric FE in the explicit FEM analysis in the ABAQUS 6.11 software and the modeling of ultrasonic wave propagation in the standard FEM analysis is impossible due to the size of the model. Therefore, instead of the PZT SA actuator, boundary conditions were used in the wave propagation models in the form of displacement perpendicular to the plane of the PZT plate equal to 1.0 × 10^−5^ (m) at the surface of 8 × 8 mm.

### 3.2. Numerical Modeling of Wave Propagation

When a part of a rigid body is excited to small oscillations, then it becomes a source of waves that, due to its elastic properties, spread through its body. With a three-dimensional rigid body, the wave propagates undisturbed in all directions until it reaches the end of the body, contact surfaces with other bodies, or damage. Knowledge about the wave propagation and reflection in an elastic rigid body is essential for damage detection in concrete structures.

Numerical modeling of ultrasonic wave propagation induced by piezoelectric SA actuators in concrete plates was performed using the explicit finite element method in the commercial software ABAQUS/EXPLICIT 6.11. Explicit dynamic analysis in ABAQUS/EXPLICIT software is a direct iterative method based on the method of central differences and the use of diagonal mass matrices. The implicit or standard finite element method is very inefficient for modeling wave propagation, especially in the ultrasonic domain.

Equation (9) represents the dynamic equilibrium equation:

(9) $$M\ddot{U}+C\dot{U}+KU=F_{a}$$

where $\ddot{U},\;\dot{U}\;\mathrm{and}\;U$ are acceleration, velocity and displacement vectors, respectively. M—diagonal mass matrix, C—damping matrix, K—stiffens matrix and $F_{a}$—load vector.

Rayleigh damping can be defined in relation to stiffness and mass as follows:

(10) $$C=\alpha_{R}M+\beta_{R}K$$

where $\alpha_{R}$ and $\beta_{R}$ are damping coefficients proportional to mass and stiffness or Rayleigh damping factors. In general, the mass-proportional coefficient ($\alpha_{R}$) attenuates lower frequencies, while a coefficient proportional to stiffness ($\beta_{R}$) damps higher frequencies. Coefficient $\alpha_{R}$ introduces damping forces using absolute model accelerations, which indicates that this coefficient introduces damping as a characteristic of the structure, while coefficient $\beta_{R}$ introduces damping proportional to strain, which can be interpreted as part of the damping accepted by the material with its properties.

Equation (9) represents a system of linear differential equations of the second order and can be solved using the standard procedures of solving differential equations with constant coefficients. However, these methods are very computationally demanding, especially when the matrices are large. In solving practical problems of wave propagation modeling in 3D concrete plates, it is necessary to use efficient techniques. One of these techniques is the direct integration method (EFEM) available in the commercial software ABAQUS/EXPLICIT. In this method, Equation (9) is integrated using a step-by-step procedure. The term “direct” refers to the character of the numerical procedure, i.e., that there is no need to transform differential equations [70].

Wave propagation occurs when the initial state of equilibrium is disturbed by forces or displacements introduced into the model via nodes. Usually, the excitation is introduced into the system by means of short-time signals. The equation of the body motion is integrated using the method of central differences (Equations (11) and (12)) is as follows:

(11) $${\ddot{U}}^{\left(t\right)}=\frac{1}{\Delta t^{2}}\left(U^{\left(t-\Delta t\right)}-2U^{\left(t\right)}+U^{\left(t+\Delta t\right)}\right)$$

(12) $${\dot{U}}^{\left(t\right)}=\frac{1}{2\Delta t}\left(U^{\left(t+\Delta t\right)}-U^{\left(t-\Delta t\right)}\right)$$

where t—time and Δt—time step (increment). Using Equations (11) and (12) in Equation (9) the following equations are obtained:

(13) $$\left(\frac{1}{\Delta t^{2}}M+\frac{1}{2\Delta t}C\right)U^{\left(t+\Delta t\right)}=R^{\left(t\right)}-\left(K-\frac{2}{\Delta t^{2}}M\right)U^{\left(t\right)}-\left(\frac{1}{\Delta t^{2}}M-\frac{1}{2\Delta t}C\right)U^{\left(t-\Delta t\right)}$$

Based on Equation (13), it can be concluded that the solution for $U^{\left(t+\Delta t\right)}$ is obtained on the basis of equilibrium conditions at the time t and t−Δt, which is why the integration procedure is called explicit. In order for the calculation procedure to be possible, it is necessary to define the initial conditions in time −Δt:

(14) $$U^{\left(-\Delta t\right)}=U^{0}-\Delta t{\dot{U}}^{0}+\frac{\Delta t^{2}}{2}{\ddot{U}}^{0}$$

The explicit FEM based on central differences integrates over time using usually very small time steps Δt. An integration procedure that requires the use of a time step Δt smaller than the critical time step $\Delta t_{crit}$ is called conditionally stable. For damped systems, the condition has to be satisfied as follows:

(15) $$\Delta t\leq \Delta t_{cr}=\frac{2}{\omega_{max}\left(\sqrt{1+\xi^{2}}-\xi \right)}$$

For wave propagation modeling, where a small deformation of finite elements is assumed, the usual approximation adopted is that the critical time step represents the time of longitudinal waves take to propagate through the smallest finite element in the model as follows:

(16) $$\Delta t\leq \Delta t_{cr}=\frac{L_{e}}{c_{d}}$$

where $L_{e}$—characteristic dimension of finite element and $c_{d}$—longitudinal wave propagation speed.

For wave propagation simulation, it is necessary to use at least several finite elements per one wavelength (one of the recommendations is at least seven and for some analyses up to 40 are recommended). The higher the frequency of the wave, the shorter its wavelength and a smaller size of FE is necessary that furtherly results in a smaller time step. The very small time step further limits the explicit procedure to either very short simulations or smaller models. When modeling wave propagation using the explicit FEM, the general advices are as follows: 1. use a time step not much smaller than the critical time step, 2. make smaller and simpler numerical models on which the desired conclusions can be made, 3. use the lowest possible frequency that meets the needs damage detection. In structural engineering, this narrows down the possibilities to modeling only a relatively small laboratory samples.

## 4. Experimental and Numerical Verification of Hybrid Damage Localization Approach

**Figure 7:**
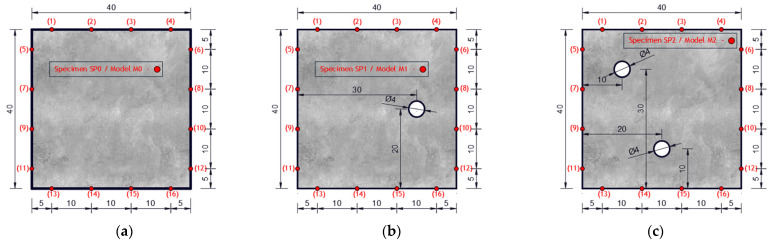
Specimen/model geometry. (**a**) Specimen SP0 and model M0, (**b**) specimen SP1 and model M1, (**c**) specimen SP2 and model M2 (lengths in cm).

Experimental investigation was performed on square concrete plates with dimensions of 40 × 40 × 4 cm. Three experimental samples were analyzed. One undamaged plate (SP0), one plate with one circular damage (SP1) and one plate with two circular damages (SP2). The diameter of the circular damage is 4 cm and their exact position is shown in Figure 7. Circular damage in all analyzed experimental samples and numerical models extends through the entire thickness of the concrete slab. Sixteen PZT A/S positions were selected. Figure 7 shows the geometry of experimental samples, with the position of the damage and the PZT A/S. The effectiveness of the hybrid method and numerical modeling approach was evaluated on these three samples.

**Figure 8:**
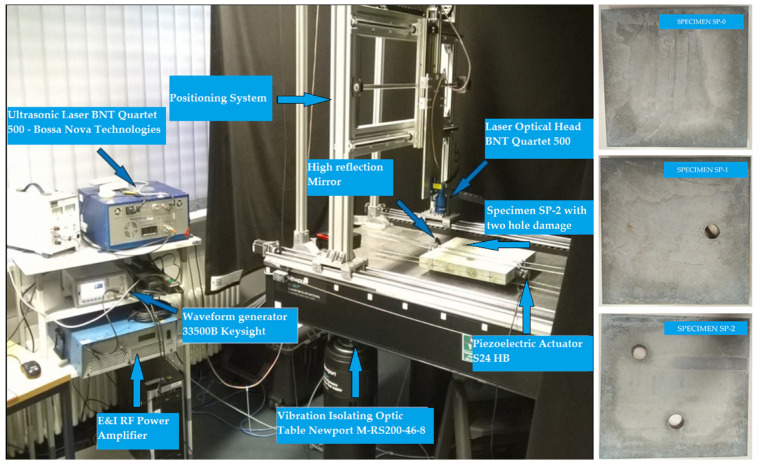
Experimental setup for the hybrid PZT-Laser scanning system. (**left**) Amplifier, waveform generator, PC computer, hardware of ultrasonic laser, concrete specimen set on a vibration-isolating optic table with PZT actuator and laser optic head and (**right**) Specimens SP0, SP1, SP2.

Concrete plates were made of concrete class C25/30 with aggregate fractions of diameters 0–4 mm, 4–8 and 8–16 mm and the ratio of application of concrete ingredients shown in Table 2. Experimental samples were made in the Laboratory for Construction Materials of the Faculty of Civil Engineering and Architecture of the University of Niš (Niš, Serbia) at a temperature of ±22 °C. In the experimental analysis, circular holes in the samples were made during the preparation of the samples through placing the circular formwork before pouring the concrete. The position of the damage (hole) was adopted relatively arbitrarily. When choosing the position, the criteria that were taken into account are as follows: 1. that the hole should not be symmetrical in relation to the position of the PZT SA, 2. that it should not be in the middle or at the very edge of the plate, 3. for models with two damages, the distance between the holes should be proportional to the size of the model (so that the holes are not too close or far away). The measurements were made in the Laboratory for Mechanics of Adaptive Systems at the Ruhr University in Bochum (Germany). For the purposes of experimental verification, piezoelectric actuator S24 HB^®^ (manufactured by Karl Deutsch, Wuppertal, Germany) and a 3.5-cycle Hanning windowed tone-burst input signal with a central frequency of 100 kHz and a duration of 3.5·10^−5^ (s) were used to excite the structure instead of PZT SAs. The obtained wavelength $\lambda_{w}=35.8\;mm$ was smaller than the size of the damage and larger than the largest aggregate grain. The ultrasonic laser scanner BNT Quartet 500^®^, manufactured by Bossa Nova Technologies (Los Angeles, CA, USA), was used as a sensor. The system included the ability to position the optical laser and the sample was placed on an optical table to isolate external vibrations (Newport M-RS200-46-8^®^, Newport Beach, CA, USA) Figure 8.

**Figure 9:**
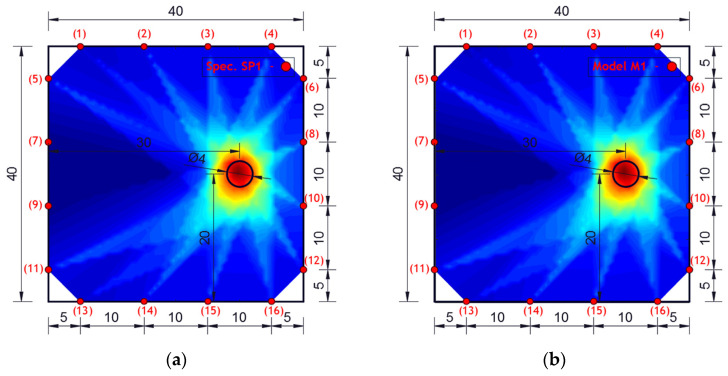
Damage localization using a hybrid approach for specimen/model. (**a**) Specimen SP1 and (**b**) model M1 (lengths in cm).

The measurement procedure includes the following steps of 1. fixing the PZT actuator S24 HB for the concrete sample at the exact position, 2. the placement of the optical laser at the place of the sensor together with the reflecting mirror, 3. the emission of a mechanical wave by the PZT actuator and the detection of incoming wave by the sensor, 4. moving the sensor to the next location and repeating the procedure. A total of 100 output signals were measured for each sensor position and statistical processing of the signals was performed. The wave propagation speed was measured, the Rayleigh damping coefficients were calculated and the following results were obtained of ($C_{Lexp}=3578.43\;m/s,$ $\alpha =2058.23\;and\;\beta =1.105\times 10^{-8}$). Based on the experimentally obtained output signals, the hybrid method was applied and the results are shown in Figure 9.

The experimentally investigated samples were numerically simulated using the explicit FEM using ABAQUS/EXPLICIT software. 3D models were created for the numerical simulation of wave propagation in concrete plates using C3D8R FE. A linear elastic model with Rayleigh damping was used in order to account for the concrete microstructure as shown in Table 1.

**Figure 10:**
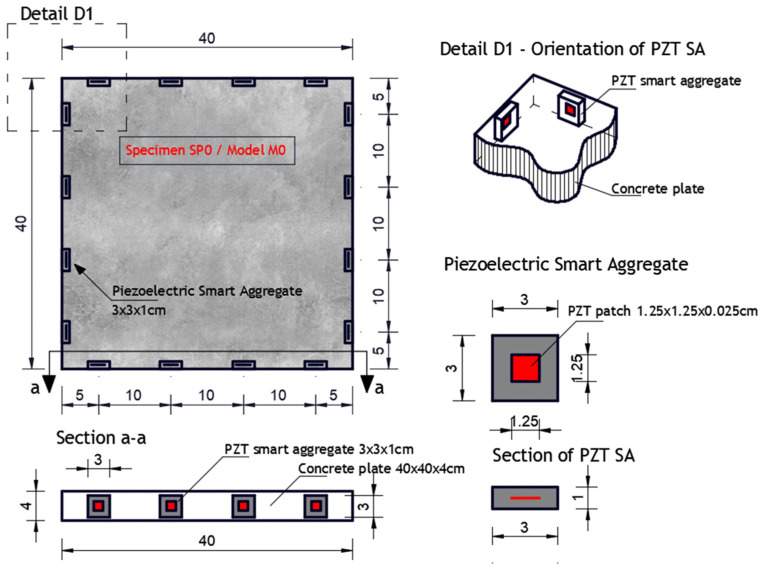
Numerical model M0. Position of PZT SAs, orientation and dimensions, (lengths in cm).

Figure 10 shows the geometry of the numerical model with the position and number of PZT SAs applied in this research. The dimensions of the concrete slabs were 40 × 40 × 4 cm. Sixteen PZT SAs with the dimensions of 3 × 3 × 1 cm were applied. Each PZT SA consisted of a concrete block and one PZT patch with the dimensions of 1.25 × 1.25 × 0.025 cm. The orientation and exact position of PZT SAs is shown in Figure 10. The volume fraction of one PZT SA in the volume of the concrete slab was 0.14%, while the volume fraction of all the PZT SAs in the volume of the concrete slab was 2.25%.

The explicit dynamic step is defined through linear and quadratic volumetric viscosity parameters with a time duration of 2 × 10^−3^ (s). The piezoelectric actuator S 24HB was modeled using displacement boundary conditions at the point of contact between the PZT patch and the surrounding concrete. The displacement field is defined perpendicular to the PZT patch on the sides of the concrete plate at the place where the actuators are located, with the same input signal as that in the experimental investigation. The output signal obtained numerically represents the measured displacement perpendicular to the side of the concrete plate at the sensor location.

**Figure 11:**
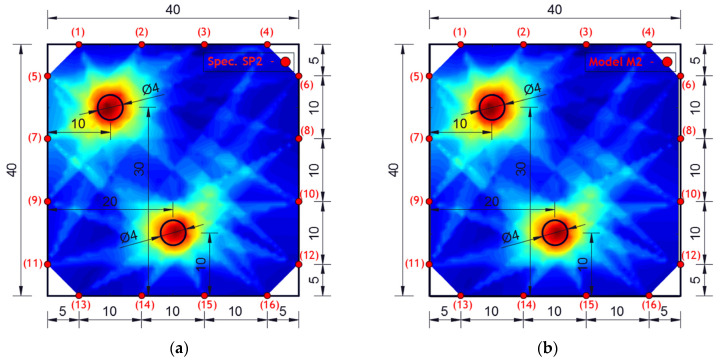
Damage localization using the hybrid approach for specimen/model. (**a**) Specimen SP2 and (**b**) model M2 (lengths in cm).

The results of damage localization obtained experimentally and numerically are shown in Figure 9 and Figure 11. The dark blue color in Figure 9 and Figure 11 indicates the lowest values of the cumulative damage index expressed in Equation (3) (DI_p_). The red color represents the highest values, while the yellow color indicates the average values of the cumulative damage index. The highest DI_p_ values indicate the position of damage in the concrete plate.

From the obtained images, it can be concluded that the agreement of the obtained results is very high—the similarity of the images is almost complete. In addition, the hybrid approach very successfully localized the damage for both single-damage and dual-damage models. A sample/model with one damage showed a complete match between the obtained image of the damage and the real location and shape of the damage. For the sample/model with two damages, the damage localization was done very successfully, but the red zone representing the damage deviates a bit more from the real circular shape of the damage. Certainly, based on the experimental and numerical results obtained, it can be concluded that it is possible to further analyze other forms of concrete plates with numerical models.

## 5. Implementation of Hybrid Damage Localization on Arbitrary Shaped Concrete Plate

**Figure 12:**
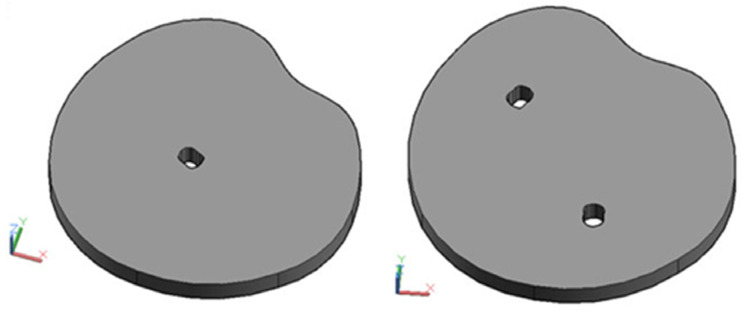
3D models prepared using CAD software (AutoCAD 2016). **Left**—model with one damage and **right**—model with two damages.

The hybrid approach (presented in Section 2) for damage localization in concrete plates will be analyzed for the flat concrete plate elements of an arbitrary shape. The verification of the developed approach for this case was performed numerically. The numerical simulation of wave propagation was performed using the software ABAQUS/EXPLICIT as described in Section 3. The specific requirements of the arbitrary shape modeling was carried out using computer-aided design (CAD) technology as shown in Figure 12.

**Figure 13:**
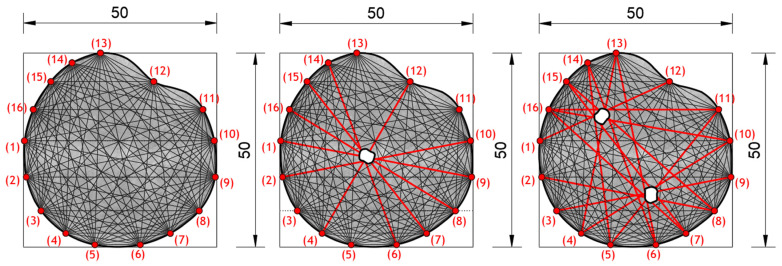
Model geometry and position of PZT SAs A/S. **Left**—Model MAR 0, **middle**—Model MAR 1 and **right**—Model MAR 2.

The three models that were analyzed are as follows: one without damage (MAR 0), one with one damage (MAR 1) and one with two damages (MAR 2). Their shapes are shown in Figure 13. Damage localization was performed using 16 PZT actuators/sensors. The position of the PZT A/S is defined arbitrarily; however, when placing the A/S, care was taken to ensure that mutual distance was approximately equal and to satisfy all the remarks given in the theoretical part of the hybrid approach. The exact position of each PZT A/S in relation to the local coordinate system XY is given in Table 3. The following model notation is defined as follows: MAR 0—represents a model without damage, MAR 1—a model with one damage and MAR 2—a model with two damages.

The geometry of the concrete body was modeled using CAD technology utilizing the “spline” and “extrude” functions in 3D Auto CAD 2016 software. The concrete body is modeled at a scale of 1:1 where one unit in the CAD program is equal to 1 m. The CAD model of the concrete slab is then exported to a SAT (Standard ACIS Text) format that is imported into ABAQUS/EXPLICIT as part of the model. Boundary conditions are defined through four points in its lower part. The excitation is undertaken in the same way as carried out in the quadratic models shown in the previous chapter. Mesh was created with finite elements that were 2 mm in size.

**Figure 14:**
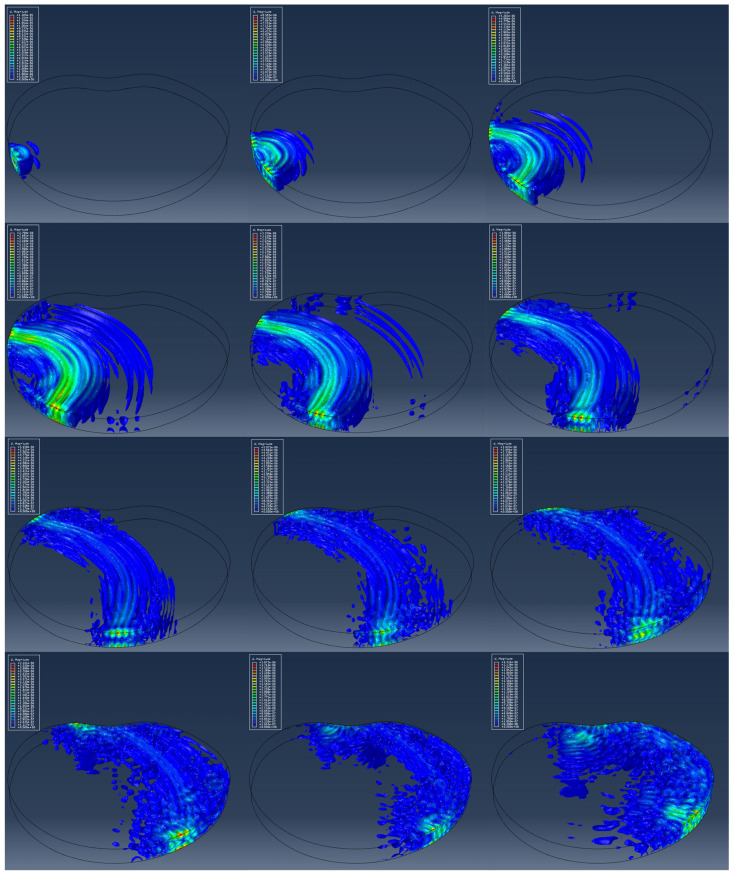
Iso-surface contour plots of the wave field for model MAR 0 generated by PZT SA actuator A1 at different time instants.

Figure 14 shows the wave propagation obtained on the basis of the numerical model. Wave propagation is illustrated through 12 screenshots at time points starting from 2.0 × 10^−5^ (s) to 2.4 × 10^−4^ (s) with a step of 2.0 × 10^−5^ (s). The representation of wave propagation through an irregularly shaped concrete structure was carried out using iso-surface contour options. Compared with standard the “banded plot” display in the ABAQUS 6.11 software, the “iso-surface contour” plot gives a better visualization of the wave propagation through the concrete slab.

**Figure 15:**
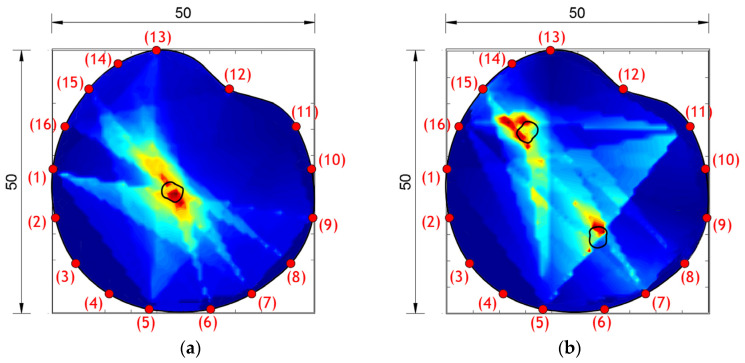
Damage localization using the hybrid approach for models with arbitrary shapes. (**a**) Model MAR 1 and (**b**) model MAR 2.

Figure 15 shows the damage localization results obtained using the hybrid approach for the analyzed arbitrary shapes of concrete plates and cases of damage. It can be seen that the highest values of the DI (red and yellow zones) appear at the places of damage, shown through the two-dimensional image generated by the MATLAB program. Based on the obtained damage images, it can be concluded that the hybrid approach can detect and localize damage even for irregularly shaped concrete plates. In comparison with the results obtained on square plates, it can also be concluded that the success of damage localization with arbitrary plates is somewhat inaccurate.

**Figure 16:**
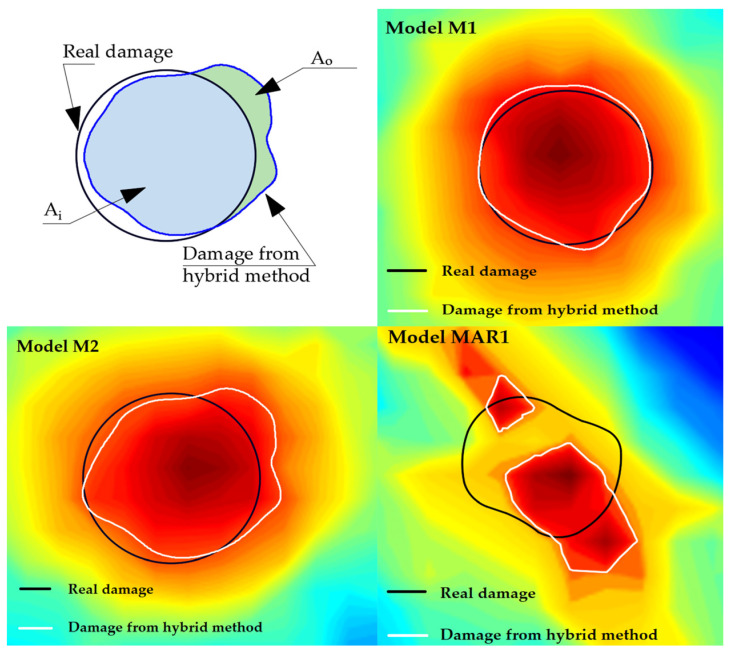
Surfaces (A_r_, A_h_, A_o_, and A_i_) and obtained results for the models M1, M2 and MAR1.

For the purposes of the quantitative assessment of detection, localization and the quantification of damage, three new parameters of a_1_, a_2_ and a_3_ given in Equation (17) were introduced:

(17) $$a_{1}=\frac{A_{i}}{A_{r}}\cdot 100\%;\;a_{2}=\frac{A_{o}}{A_{i}}\cdot 100\%;\;a_{3}=\left\{\begin{array}{c} \frac{A_{r}}{A_{h}}\cdot 100\%,\left(A_{r}<A_{h}\right)\;\\ \frac{A_{h}}{A_{r}}\cdot 100\%,\left(A_{r}>A_{h}\right) \end{array}\right.$$

where $A_{r}$—area of real damage, $A_{h}$—area of damage obtained by the hybrid approach, $A_{i}$—area of union of real damage and damage shape obtained through the hybrid method for damage localization (Figure 16) and $A_{o}$—area of difference of the damage shape obtained through the hybrid method for damage localization and real damage (Figure 16).

Figure 16 shows the areas of $A_{i}$ and $A_{o}$ as well as the obtained shapes for models M1, M2 and MAR1. Table 4 shows the obtained results of the parameters for the quantitative assessment of the detection, localization and quantification of damage for all models and experimental samples.

When evaluating the quality of the obtained results, the rules apply as follows: (a) the results are better if coefficients a_1_ and a_3_ are closer to 100% and (b) the opposite rule applies to coefficient a_2_, that is, the best results are obtained when the coefficient is as close as possible to zero.

## 6. Conclusions

This paper presents the application of a hybrid approach based on the energy of wave propagation for damage localization in concrete plates of arbitrary shape and constant thickness. First, the hybrid algorithm was experimentally analyzed on square concrete slabs with one or two damages. The numerical simulation of the PZT SA actuator was performed using standard FEM analysis and wave propagation simulation in square concrete plates using the explicit FEM. The results obtained on the basis of numerical simulations using the ABAQUS 6.11 software match well with the experimentally obtained results, which verifies the applied numerical procedure. The presented numerical procedure for modeling PZT SAs and wave propagation using the IFEM and the EFEM is an effective and practical tool for further research. The verification of the numerical simulation was undertaken based on the comparison of the final damage images obtained using the hybrid approach.

Table 5 summarized the results of this research. In total, two experimental samples, SP1 and SP2, and four numerical models were analyzed. The success of the damage detection, localization and quantification was evaluated using the descriptive terms of (a) successful, (b) partially successful and (c) unsuccessfull.

Finally, considering that the hybrid approach was developed to be able to localize damage for an arbitrary shape of a concrete structure, numerical verification was carried out on models of concrete plates with an arbitrary shape. The three models that were analyzed are as follows: a model without damage, one with one damage and one with two damages, with a total of 16 positions of PZT SAs A/S. Damage localization results were obtained with satisfactory accuracy. Based on the damage images obtained using the hybrid algorithm, it can be seen that the position of the maximum values of the damage index is in the specified damage zones with certain deviations. Based on the visual comparison of the images obtained using the hybrid approach and the real shape of the damage, a great match of the results can be observed. In addition, the parameters for the quantitative assessment of detection, localization and quantification of damage, which give more detailed information about the obtained results, were calculated and shown in Table 4. Based on the obtained parameters, it can be concluded that the best match between the real damage and the one obtained based on the hybrid approach is observed in the case of model M1 and sample SP1 with one damage (the value of the coefficients for the M1 model is as follows: a_1_ = 96.18%, a_2_ = 7.27%, a_3_ = 96.84%, while the values of the experimental sample SP1 are as follows: a_1_ = 94.72%, a_2_ = 8.14%, a_3_ = 95.13%,). Furthermore, based on the parameter values, it can be concluded that the numerical results are very close to the experimental ones. Their significant match indicates a good numerical modeling approach. The M2 model and the SP2 sample with two defects have slightly lower results than the one damage model; however, the success rates of damage detection, localization and quantification are still very high.

In the case of arbitrary shape models, the situation is significantly worse in terms of damage quantification. The general location detected using the hybrid method is visually very close to real damage, but the form of damage generated using the method has a significantly lower percentage of similarity to real damage. The following coefficient results were obtained for the MAR1 model: a_1_ = 46.52%, a_2_ = 85.71%, a_3_ = 69.94%. With the MAR2 model, the percentages are even more unfavorable. Based on the obtained results for models with an irregular shape, it can be concluded that the presented hybrid method can detect damage but cannot quantify damage in a high percentage of success.

For all the analyzed models/samples, the damage is spread over the entire thickness of the concrete slab and the obtained results are presented in this paper. Given that in real structures the damage can be spread through only a part of the thickness of the concrete slab, future research will be focused on applying the proposed method to that type of damage.

The presented numerical modeling procedure as well as the hybrid algorithm for damage localization can be further used with sufficiently good reliability for different combinations of concrete plate geometries, numbers of PZT SAs A/S and their positions.

## Figures and Tables

**Table 1:** Material characteristics of a PZT patch and concrete for FEM analysis.

PZT Material Properties					
Density (kg/m^3^)	Dielectric Properties (CV^−1^/m) · 10^−8^				
ρ = 7500	D_11_ = 1.505		D_22_ = 1.301		D_33_ = 1.505
Piezoelectric properties (F/m) · 10^−10^					
d_111_ = 0	d_122_ = 0	d_133_ = 0	d_112_ = 7.41	d_113_ = 0	d_123_ = 0
d_211_ = −2.74	d_222_ = 5.93	d_233_ = −2.74	d_212_ = 0	d_213_ = 0	d_223_ = 0
d_311_ = 0	d_322_ = 0	d_333_ = 0	d_312_ = 0	d_313_ = 0	d_323_ = 7.41
Material characteristics—Engineering constants					
E_1_ = 60.61 GPa	E_2_ = 48.31 GPa	E_3_ = 60.61 GPa	ν_12_ = 0.512	ν_13_ = 0.289	ν_23_ = 0.408
G_12_ = 23.0 GPa		G_13_ = 23.5 GPa		G_23_ = 23.0 GPa	
Concrete Material Properties					
Young’s modulus of elasticity (GPa)	Poisson’s ratio	Rayleigh damping coefficients		Density (kg/m^3^)	
44.30	0.15	α = 2050 (1/s)	β = 1.10 · 10^−8^ (s)	2400	

**Table 2:** Experimental concrete samples—concrete ingredients and density.

Marks of Experimental Samples—SP_i_	Weight—W_i_ (kg)	Density—ρ_i_ (kg/m^3^)	Mean Value ρ_mean_ (kg/m^3^)	Deviation $\left|\frac{\rho_{i}-\rho_{mean}}{\rho_{i}}\right|$ · 100(%)
Plate without damage: SP0	15.292	2389.375	2377.3	0.505
Plate with one hole: SP1	15.068	2373.025		0.180
Plate with two damages: SP2	14.982	2378.280		0.040
Concrete ingredients	Fine-grained sand (kg/m^3^)	Coarse-grained sand (kg/m^3^)	Cement (kg/m^3^)	Water (kg/m^3^)
	685.00	1135.00	405.00	182.00

**Table 3:** XY coordinates of the PZT actuators/sensors.

Number of PZT Actuators/Sensors for Models with Arbitrary Shape																
	1	2	3	4	5	6	7	8	9	10	11	12	13	14	15	16
Models with 16 PZT actuators/sensors—XY coordinates																
X	0.21	0.69	4.53	10.89	18.47	30.16	37.96	45.37	49.59	49.34	46.4	33.7	19.87	12.56	7.03	2.49
Y	27.36	18.04	9.34	3.52	0.58	0.58	3.52	9.34	18.04	27.36	35.46	42.01	50.0	47.47	42.61	35.46

**Table 4:** Parameters for the evaluation of detection, localization and quantification of damages (a_1_, a_2_ and a_3_).

	M1	M2		SP1	SP2		MAR1	MAR2	
a_1_ (%)	96.18	89.18	88.92	94.72	90.63	91.18	46.52	20.57	30.05
a_2_ (%)	7.27	17.39	17.76	8.14	15.75	15.22	85.71	98.46	200.0
a_3_ (%)	96.84	92.43	92.07	95.13	93.12	92.95	69.94	40.82	90.30
A_r_ (cm^2^)	12.57	12.57	12.57	12.57	12.57	12.57	6.32	6.32	6.29
A_h_ (cm^2^)	12.98	13.16	13.65	13.21	13.50	13.52	4.42	2.58	5.68
A_i_ (cm^2^)	12.09	11.21	11.17	11.91	11.39	11.46	2.94	1.30	1.89
A_o_ (cm^2^)	0.89	1.95	2.47	1.30	2.11	2.06	2.52	1.28	3.78

**Table 5:** Summarized results of the experimental and numerical analysis of damage detection, localization and quantification.

Marks	Analysis	Dimensions (cm)	Number of PZT SAs	Damage Detection	Damage Localization	Damage Quantification
SP1	Experimental	40 × 40 × 4	16	Successfull	Successfull	Successfull
SP2	Experimental	40 × 40 × 4	16	Successfull	Successfull	Successfull
M1	Numerical	40 × 40 × 4	16	Successfull	Successfull	Successfull
M2	Numerical	40 × 40 × 4	16	Successfull	Successfull	Successfull
MAR 1	Numerical	Arbitrary	16	Successfull	Partially successfull	Partially successfull
MAR 2	Numerical	Arbitrary	16	Successfull	Partially successfull	Partially successfull

## Data Availability

The author’s original results were used in writing this paper.
